# Parallel in vivo and in vitro transcriptomics analysis reveals calcium and zinc signalling in the brain as sensitive targets of HBCD neurotoxicity

**DOI:** 10.1007/s00204-017-2119-2

**Published:** 2017-11-25

**Authors:** V. Reffatto, J. D. Rasinger, T. S. Carroll, T. Ganay, A.-K. Lundebye, I. Sekler, M. Hershfinkel, C. Hogstrand

**Affiliations:** 1 Diabetes and Nutritional Sciences Division, King’s College London, Franklin-Wilkins Building, 150 Stamford Street, London, SE1 9NH UK; 2 Present Address: National Institute of Nutrition and Seafood Research (NIFES), Nordnes, PO Box 2029, 5817 Bergen, Norway; 3 Department of Morphology, Ben Gurion University of the Negev, POB 653, Beer-Sheva, Israel; 4 Present Address: Department of Ophthalmology and Visual Sciences, The University of Texas Medical Branch, 301 University Blvd, Galveston, TX 77555-1106 USA; 5 Present Address: MRC Clinical Sciences, Bioinformatics Centre, Imperial College London, Hammersmith Hospital, Du Cane Rd, London, UK; 6 Department of Physiology and Cell Biology, Faculty of Health Science, and Zlotowski Center for Neuroscience, Ben-Gurion University of the Negev, POB 653, Beer-Sheva, Israel

**Keywords:** BFR, Neurotoxicity, Transcriptomics, Glutamate, Oestrogen, Androgen, Dihydrotestosterone, Prolactin, GnRH

## Abstract

Hexabromocyclododecane (HBCD) is a brominated flame retardant (BFR) that accumulates in humans and affects the nervous system. To elucidate the mechanisms of HBCD neurotoxicity, we used transcriptomic profiling in brains of female mice exposed through their diet to HBCD (199 mg/kg body weight per day) for 28 days and compared with those of neuronal N2A and NSC-19 cell lines exposed to 1 or 2 µM HBCD. Similar pathways and functions were affected both in vivo and in vitro, including Ca^2+^ and Zn^2+^ signalling, glutamatergic neuron activity, apoptosis, and oxidative stress. Release of cytosolic free Zn^2+^ by HBCD was confirmed in N2A cells. This Zn^2+^ release was partially quenched by the antioxidant *N*-acetyl cysteine indicating that, in accordance with transcriptomic analysis, free radical formation is involved in HBCD toxicity. To investigate the effects of HBCD in excitable cells, we isolated mouse hippocampal neurons and monitored Ca^2+^ signalling triggered by extracellular glutamate or zinc, which are co-released pre-synaptically to trigger postsynaptic signalling. In control cells application of zinc or glutamate triggered a rapid rise of intracellular [Ca^2+^]. Treatment of the cultures with 1 µM of HBCD was sufficient to reduce the glutamate-dependent Ca^2+^ signal by 50%. The effect of HBCD on zinc-dependent Ca^2+^ signalling was even more pronounced, resulting in the reduction of the Ca^2+^ signal with 86% inhibition at 1 µM HBCD. Our results show that low concentrations of HBCD affect neural signalling in mouse brain acting through dysregulation of Ca^2+^ and Zn^2+^ homeostasis.

**Electronic supplementary material:**

The online version of this article (doi:10.1007/s00204-017-2119-2) contains supplementary material, which is available to authorized users.

## Introduction

1,2,5,6,9,10-Hexabromocyclododecane (HBCD) is a brominated flame retardant (BFR), mainly used in thermal insulation foams in building and construction, as well as in plastics and textiles. High quantities were used due to bans on many other BFRs, especially in Europe (Birnbaum and Staskal 2004; Chain EPoCitF 2012; Law et al. 2005). In 2013 HBCD was listed in Annex A (POP for elimination) under the Stockholm convention on persistent organic pollutants (POPs), with specific exceptions. Namely, HBCD can be still produced and used for expanded polystyrene and extruded polystyrene in buildings.

HBCD has been detected in the environment worldwide. It has been found in samples of urban and rural air (Covaci et al. 2006; Vorkamp et al. 2012), soil (Drage et al. 2016; Wu et al. 2016a), water (de Wit et al. 2010), and household dust (de Wit et al. 2012; Schreder and La Guardia 2014). HBCD is a lipophilic compound (log *K*_ow_ = 5.81) (American Chemistry Council 2001) that bioaccumulates through food webs, and concentrations are particularly high in oily fish, such as salmon (Chain EPoCitF 2012; Fromme et al. 2016). The main sources of human exposure to HBCD are contaminated food, breast milk, and inhalation from polluted indoor dust and air (Chain EPoCitF 2012; Fromme et al. 2016). Presence of HBCD in human breast milk has been identified in samples from various countries including Canada, United States, Europe and China. HBCD concentrations in breast milk increased from 1980 to 2002, but have reached a stable level since (Eljarrat et al. 2009; Fängström et al. 2008; Ryan and Rawn 2014; Shi et al. 2013). HBCD has been also measured in human serum (Rawn et al. 2014b; Roosens et al. 2009) and human foetal liver and placental tissues (Rawn et al. 2014a).

HBCD has the potential to cause histological changes in liver, thymus and thyroid (Maranghi et al. 2013; van der Ven et al. 2006). It can induce adverse effects to the reproductive, endocrine, and central nervous systems (Chain EPoCitF 2012; Maranghi et al. 2013; Rasinger et al. 2014; van der Ven et al. 2006, 2009). Notably, 28-day oral exposure to HBCD increased thyroid weight and activity of the thyroxine (T4) metabolising enzyme T4-UGT, and decreased total circulating thyroxin in female Wistar rats (van der Ven et al. 2006). These effects are accompanied by evidence of suppressed thyroid hormone receptor-mediated transcription (Ibhazehiebo et al. 2011). Along with effects on the thyroid hormone system, the most sensitive end-points in animal studies have related to neurodevelopment (Chain EPoCitF 2012).

Due to its lipophilicity, HBCD can pass the blood brain barrier (BBB) and accumulate in the brain (Covaci et al. 2006; Rasinger et al. 2014). Its ability to cause neurotoxicity has been evidenced by developmental behavioural effects seen after exposure (Eriksson et al. 2006; Lilienthal et al. 2009). In mice exposed to HBCD, effects on memory and learning have been reported at single oral doses between 0.9 and 13.5 mg/kg body weight (Eriksson et al. 2006), and in rats exposed to HBCD, effects on dopamine-dependent behaviour and hearing function were observed (Lilienthal et al. 2009). These effects may be related to a reduction in expression of the striatal dopamine transporter and vesicular monoamine transporter 2, as observed in studies in C57BL/6J mice (Genskow et al. 2015). However, HBCD has also been shown to inhibit dopamine uptake with a half maximal inhibitory concentration (IC_50_) of 4 µM (Mariussen and Fonnum 2003). In the same study HBCD was also seen to inhibit glutamate uptake at concentrations as low as 1 µM, but it never achieved more than 50% inhibition (Mariussen and Fonnum 2003). In addition, accumulation of xenobiotics in the brain has been seen to cause a chronic inflammatory response which has been found to be linked with neurodegenerative conditions such as Alzheimer’s and Parkinson diseases (DeLegge and Smoke 2008). In vivo studies in mice have shown that while exposure to BFRs during gestation may lead to congenital effects (Curran et al. 2011), later exposure during brain development can cause disturbed spontaneous behaviour in adults (Alm et al. 2008; Eriksson et al. 2006; Viberg et al. 2003).

In spite of the valuable information on the effects of HBCD on dopaminergic and glutamatergic neuronal function, knowledge of intracellular signalling pathways modulated by HBCD in neurons is limited. One such mechanism is likely interference with cellular Ca^2+^ homeostasis that is crucial for learning and memory function. HBCD (2–200 nM) was reported to reduce cytosolic free Ca^2+^ concentrations and increase Ca^2+^ content of the sarcoplasmic reticulum in rat H9C2 cardiomyocyte cell line (Wu et al. 2016b). In PC12 cells, 2 µM HBCD inhibited depolarisation-evoked [Ca^2+^]_i_ transients and associated catecholamine release (Dingemans et al. 2009). HBCD also inhibited the sarcoplasmic-endoplasmic reticulum Ca^2+^-ATPase (SERCA) in neuroblastoma SH-SY5Y cells through interference with its ATP binding at an IC_50_ of 2.7 µM (Al-Mousa and Michelangeli 2014). As both pre- and post-synaptic events are dependent on Ca^2+^ signalling, this mechanism of action could have widespread consequences for neuronal function.

We sought to take an unbiased approach to explore molecular events leading to neurotoxicity of HBCD in mouse, integrating in vivo and in vitro experiments. This allowed us to explore mechanisms of action indicated in the animal experiment and to differentiate between direct effects of HBCD on the brain from potential indirect endocrine effects, caused by disruption of sex steroids (Rasinger et al. 2014) and thyroid hormones (van der Ven et al. 2006). We used transcriptomics to interrogate HBCD responses on gene expression in mouse brain in vivo as well as in two neuronally derived mouse cell lines, N2A and NSC19. Post-transcriptomics hypothesis driven experimentation on cell lines and isolated primary hippocampal cells demonstrated pronounced HBCD disruption of Ca^2+^ and Zn^2+^ homeostasis.

## Materials and methods

### Animal experiment, tissue sampling and HBCD analysis

Juvenile female BALB/c mice (*n* = 12, average weight 13–15 g) were purchased from Charles River (UK). A total of six mice (3 mice per cage) were exposed to a nominal dose of 199 mg/kg body weight per day HBCD through the diet; another six mice were fed an un-spiked control diet. The animal experiment was carried out under a project licence in accordance with the UK Home Office Animals (Scientific Procedures) Act, 1986 and was performed as previously described by us (Maranghi et al. 2013; Rasinger et al. 2014). The experimental diet used was in accordance with the standard rodent diet formulation AIN-93 G with the exception that freeze-dried Atlantic salmon (*Salmo salar*) was used as the main protein and fat source. The salmon used in the present work was raised specifically for the study on plant and vegetable oil substituted fish feed, which yielded exceptionally low background levels of contaminants in the fish (Berntssen et al. 2010). A technical mixture of HBCD (1.3 g/kg feed, purity: 99.2%; Applied Biosystems, UK) was dissolved and diluted in 100% dimethylsulfoxide (DMSO) and mixed into the feed at a concentration of 1.3 mg/kg feed. The control diet was adjusted to the same final DMSO content (0.4 mL/kg feed) as the HBCD spiked feed. This dietary concentration resulted in a daily dose of 199 mg/kg body weight and was in an identical experiment found to give an accumulation of HBCD within the brain tissue of 4.7 µg/g dry weight (Rasinger et al. 2014), which on a wet weight basis corresponds to approximately 7.3 µmol/kg. This dose did not affect feed consumption or weight gain and there were no overt changes in behaviour (data not shown). After 28 days of exposure, brains were sampled and prepared for analysis as described before (Rasinger et al. 2014). In short, the whole brain was excised from the cranial cavity and dissected on ice. Left cortices were flash-frozen in liquid nitrogen and ground in a liquid nitrogen cooled mortar and pestle. Frozen powdered tissue was then partitioned in aliquots and stored at − 80 °C.

### RNA extraction and transcriptomic analysis of murine juvenile brains

Total RNA was extracted from powdered brain tissue of HBCD exposed and control mice (*n* = 6 per group) as described by us before (Rasinger et al. 2014). In short, total RNA was extracted using Trizol (Invitrogen, Life Technologies, USA) and the Qiagen RNeasy-Mini kit (Qiagen, Canada). RNA purity was assessed using a Nanodrop ND-100 UV–Vis Spectrophotometer (Nanodrop Technologies, USA) and RNA quality and integrity were assessed using the Agilent 2100 Bioanalyzer in combination with the RNA 6000 LabChip kit (Agilent Technologies, USA). Gene expression microarray analysis was performed using GeneChip Mouse Exon 1.0 ST arrays (Affymetrix, USA) following the manufacturer’s instructions. Array images were acquired using a GeneChip Scanner 3000 (Affymetrix, USA). Array data were normalised using RMAsketch as implemented in the Affymetrix Expression Console.

### Primary mouse hippocampal culture

Primary hippocampal cultures were obtained from postnatal P0-P2 days old mice that were anesthetised with isoflurane according to approved protocols. Hippocampi were removed and immediately placed in Hank’s balanced salt solution with HEPES (20 mM) and triturated in plating medium consisting of Neurobasal-A medium supplemented with 5% defined FBS, 2% B27, 2 mM Glutamax I, and 1 μg/mL gentamicin. Cells were then seeded on Poly-d lysine coated glass coverslips, in plating medium. 48 h following plating medium was replaced by serum-free culture medium that consisted of Neurobasal-A, 2 mM Glutamax I, and 2% B27.

### Cell line culture

N2A and NSC19 cells were kindly provided by Prof. Joerg Bartsch (Philipps-Universität, Marburg, Germany). Both the cell lines were cultured in DMEM with 110 mg/sodium pyruvate, supplemented with 100 units/mL penicillin, 100 units/mL streptomycin, and 10% fetal bovine serum (FBS). Cells were cultured at 37 °C in a humidified atmosphere with 5% CO_2_.

### MTT assay

To test the cytotoxicity of HBCD, cells were seeded into 96-well plates in 100 µL of DMEM. After 24 h of cell attachment, the cells were exposed to a series of HBCD concentrations. Serial stock solutions of HBCD were prepared in DMSO; the final concentration of DMSO in HBCD-containing medium was below 0.1%, which is well tolerated by neuroblastoma cells (Xia et al. 2008). Control wells received 0.1% DMSO only. To perform a (3-(4,5-dimethylthiazol-2-yl)-2,5-diphenyltetrazolium bromide (MTT, SIGMA, UK) colorimetric assay, cells seeded into 96-well plates (density: 1 × 104 cells/well) were exposed to HBCD at concentrations between 1.56 and 50 µM for 48 h. Following HBCD exposure, the medium was removed and 100 µL of the MTT salt diluted in fresh media at a concentration of 0.5 mg/mL were added to each well. Cells were incubated for 4 h in incubator at 37 °C and 5% CO_2_. After incubation, the MTT salt was replaced by 20 µL of DMSO to dissolve the purple formazan. The absorbance was measured spectrophotometrically at a wavelength of 570 nm. The background absorbance was measured at 650 nm and was subtracted from the first measurement.

### Caspase-3 assay

Caspase-3 activity was measured as a marker of apoptosis using a commercial kit (Caspase-3 assay, Invitrogen, UK). Cells seeded into a 12-well plate (density of 1 × 10^6^ cells/well) were exposed to HBCD at four different concentrations: 1, 2, 4 or 8 µM for 24 h. After HBCD treatments, cells were collected, washed twice with Phosphate-Buffered Saline (PBS, GIBCO, UK) and the Caspase-3 assay was performed as per recommendations of the supplier.

### Analysis of intracellular Zn^2+^

Free and loosely bound Zn^2+^ was detected in the neuroblastoma N2A cell line using the zinc-specific fluorescent dye Zynpyr-1 (Mellitech, France; *K*_d_ = 0.7 ± 0.1 nM). Zinc assays were performed in cells grown in a 96-well plate, detecting the fluorescence with a fluorescence plate reader. Cells were exposed to HBCD at different concentrations for 24 h.

After treatments on coverslips or in a 96-well plate, cells were washed and kept moisturised with Ringer buffer (3 M NaCl, 2.7 M KCl, 0.8 M MgCl_2_, 2 M Hepes, 0.75 M glucose, 1.8 M CaCl_2_, calibrated to pH 7.4). Zinpyr-1 dye was diluted in PBS at a concentration of 16 µM. After exposure to the dye for 30 s, cells were washed with PBS for 30 s. The fluorescence was measured with the plate reader Thermo Scientific Fluoroskan Ascent at an excitation wavelength of 480 nm and emission wavelength of 530 nm. Fluorescence emission was normalized to the DMSO control in each row separately and the Zn^2+^-induced fluorescence expressed as fold-change relative to the control.

Specificity of the zinc signal was verified by pre-treatment (2 h) with the zinc chelator diethyldithiocarbamate (DEDTC, Sigma Aldrich) at 50 µM. Alternatively, cells were pre-treated with 10 µM of the zinc chelator and antioxidant *N*-acetylcysteine (NAC) for 1 h. Activation of the NO pathway was investigated performing a 1 h treatment with 10 µM of the NO synthase blocker L-NAME before HBCD exposure.

### Ca^2+^ imaging

The intracellular concentration of free Ca^2+^ was imaged in cultured primary hippocampal neural cells. Cultures were maintained for 9–12 days before imaging and then treated with HBCD at the indicated concentrations for 24 h. For intracellular Ca^2+^ measurements, cultures were loaded with Fura-2 AM (5 µM, 20 min) and fluorescent imaging was performed as described previously, using the ratio of the emission signal following excitation with 340 nm and 380 nm (Besser et al. 2009). Representative traces of fluorescent signal changes are presented. The initial rate of the response following addition of glutamate or Zn^2+^ was determined using a linear fit, and the averaged response of at least seven slides from three different cultures is presented in the bar graphs. Since the AM dye requires hydrolysis by intracellular esterases to remain within the cell, only live neurons accumulate Fura-2 and are used for the subsequent Ca^2+^—response analysis. To determine the survival of cells following HBCD treatments, representative cultures were washed following the 24 h treatment and mounting medium containing DAPI (Santa Cruz Biotechnology) was applied. Coverslips were observed using fluorescence microscope Axioscop 2 (Zeiss) with the appropriate filters at 10 × magnification.

### RNA extraction and transcriptomic analysis of N2A and NSC19 cells

N2A and NSC19 cells were exposed to either 1 or 2 µM of HBCD, a DMSO control or a negative control (medium only). After 24 h exposure, total RNA was extracted and subjected to a custom two-colour microarray analysis (mouse OpArray, 4.0, Operon, USA) as detailed by us before (Rasinger et al. 2014). In brief, total RNA was extracted using Trizol (Invitrogen, Life Technologies, USA) and the Qiagen RNeasy-Mini kit (Qiagen, Canada). RNA purity was assessed using a Nanodrop ND-100 UV–Vis Spectrophotometer (Nanodrop Technologies, USA) and RNA quality and integrity were assessed using the Agilent 2100 Bioanalyzer in combination with the RNA 6000 LabChip kit (Agilent Technologies, USA). A cDNA pool generated from a pool of RNA of all samples was used as reference in two-colour microarray assays. Superscript III™ Indirect labelling system (Invitrogen) and Cy3 or Cy5 fluorescent dyes (Mono-Reactive Dye Pack, Amersham Biosciences) were used to create, purify and fluorescence-label aminoallyl complementary DNA (cDNA) following the manufacturer’s (Invitrogen) instructions for indirect labelling. Cy3 (reference) and Cy5 (sample) labelled cDNA were hybridised to microarrays spotted in house with 35,852 65-mer oligonucleotide probes (mouse OpArray, 4.0, Operon, USA), according to the manufacturer’s instructions. Images of hybridised slides were acquired using ScanArray^®^ Express (Perkin Elmer Inc., USA). Acquired images were interpreted using the BlueFuse (BlueGnome) software. The arrays were first evaluated to exclude spots, which were miss-aligned or in areas with high background. Spot intensities were then calculated with Bayesian background correction filtering out those with low confidence (*p* < 0.05). Prior to normalisation all genes scoring less than 0.1 in their probability of biological signal in more than 25% of arrays were removed using the Genefilter Bioconductor version 2.4 package. This filter left around 50% of total genes on the array. Loess and median scale normalisation were applied to normalise data within and between arrays, respectively.

### Transcriptomics statistics and bioinformatics

Statistical analyses of the filtered and normalized in vivo and in vitro microarray data were performed as described in Rasinger et al. (2014). In short, using the packages LIMMA and Multtest of the Bioconductor software suite (version 2.4, http://www.bioconductor.org) differential expression was identified and multiple test correction was applied to all *p* values. Genes for treatment effects were regarded as differentially expressed if the *p* value was below 0.05 and the multiple test corrected *p* value, the false discovery rate (FDR) was below 20%. Genes passing these thresholds were imported into the Ingenuity Pathway Analysis software suite and mapped onto their corresponding objects in the Ingenuity Knowledge Base (IPA, Ingenuity Systems, USA). To compare and contrast individual and common responses to HBCD exposure in gene expression, successfully mapped genes were subjected to an IPA Core Analysis (default settings) followed by an IPA Comparison Analysis (default settings). The global Ingenuity Knowledge Base (Genes Only) was used as a reference set and included endogenous chemicals; both direct and indirect relationships were included in networks that contained at least one gene from the imported list (‘‘Focus Genes’’). Only relationships based on Experimental Observations were considered. The *p* values reported for over-representation of genes in functional or pathway processes are from a right-tailed Fisher’s exact test and a *p* value cut-off of 0.05 was applied.

### Real-time quantitative PCR (qPCR)

To validate the microarray assays, synthesis of cDNA was performed using 5 µg of total- RNA previously used for microarrays. cDNA was generated using Superscript III as described by the manufacturer (Invitrogen). Primer pairs were synthesised by PrimerDesign or Qiagen (QuantiTect Primer Assay), and the sequences of primers or the Qiagen ID are shown in Supplementary Tables 1A and 1B, respectively. The assays were performed using the ABI PRISM 7700 therocycler (ABI, USA), carrying out the method as described in the SYBR Green SuperMix-UDG Kit (Invitrogen). Primer efficiency was tested and a range between 90 and 110% was considered acceptable. The housekeeping gene for qPCR normalisation was selected using GeNorm reference gene selection kit (Primerdesign), and gene Gapdh was found the least variable housekeeping gene. Quantity calculations were performed using the REST (relative expression software tool) software (Pfaffl et al. 2002). Statistical calculation of probability of differential expression were based on a randomisation of samples using the Pair Wise Fixed Reallocation Randomisation Test (Pfaffl et al. 2002). REST was set for a number of 1000 randomisations during this analysis.

## Results

### Cell viability and assessment of apoptosis

**Fig. 1:**
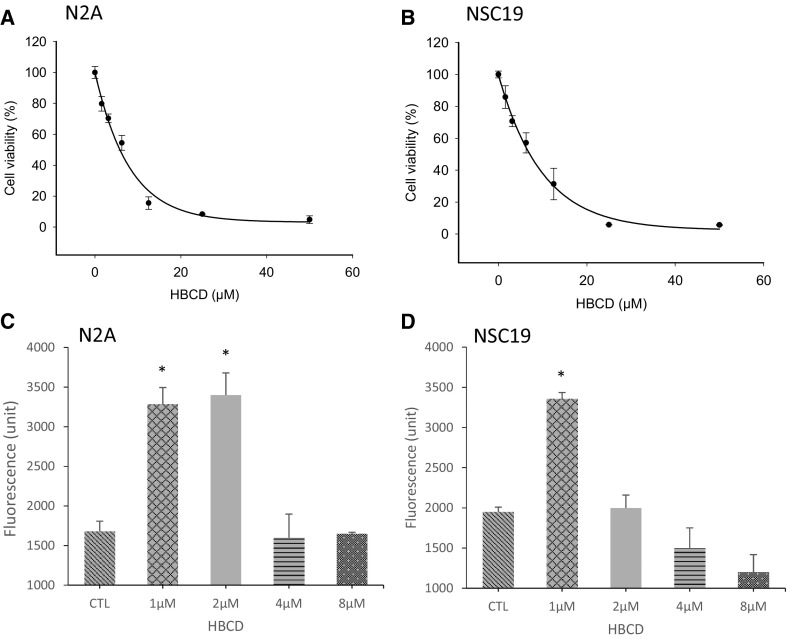
Viability of N2A and NSC19 cells exposed to HBCD: **a** N2A and **b** NSC19 cells were incubated for 48 h with a geometric series of concentration between 1.56 and 50 µM of HBCD and viability was measured with the MTT assay. Three independent experiments were performed using eight replicates in each, and the average between replicates and experiments are reported (*n* = 3) with error bars showing the standard deviation. The regression curves were fitted in SigmaPlot (Version 12), using the “Exponential Decay, Single, 3 Parameter” model. The cellular viability is express in percentage of the DMSO control. A caspase-3 assay was used as indicator of apoptosis in the **a** N2A cell line and **b** in the NSC19 cell line exposed to HBCD. The two cell cultures were incubated for 24 h with four different concentrations of HBCD (1, 2, 4 or 8 µM). A caspase-3 fluorescence assay was then performed and the fluorescence measured every 15 min in a microplate reader with 485 nm excitation and 530 nm emission, and is here expressed in arbitrary units. Three independent experiments were performed using eight replicates in each, and the average between replicates and experiments are reported (*n* = 3). Error bars denote standard deviation and an asterisk represents a *p* < 0.05

Cells exposed to HBCD at concentrations between 1.56 and 50 µM dose-dependent cytotoxicity compared to the control (Fig. 1a, b). Cell viability, as measured by the MTT assay, was reduced by about 20-30% at concentrations between 1.5 and 3.15 µM for N2A and NSC-19 cell lines, respectively. At concentrations greater than 6.25 µM, cell viability decreased by more than 50% for both cell lines and up to 100% at concentration of 12.5 µM for N2A cells and 25 µM for NSC-19 cell line. The EC50 was estimated to be about 5 and 6 µM for the N2A and the NSC19 cell lines, respectively (Fig. 1a, b).

Caspase-3 activity was measured as a marker of apoptosis in cells exposed for 24 h to HBCD at concentrations ranging from 1 to 8 µM. There was a significant (p < 0.05) increase in caspase-3 enzymatic activity at 1 and 2 µM HBCD for the N2A cell line (Fig. 1c) and at 1 µM for the NSC-19 cell line (Fig. 1d). At concentrations higher than these, the caspase-3 activity showed no difference compared to the control, presumably because of the loss of viable and functional cells.

### Microarray experiments

**Fig. 2:**
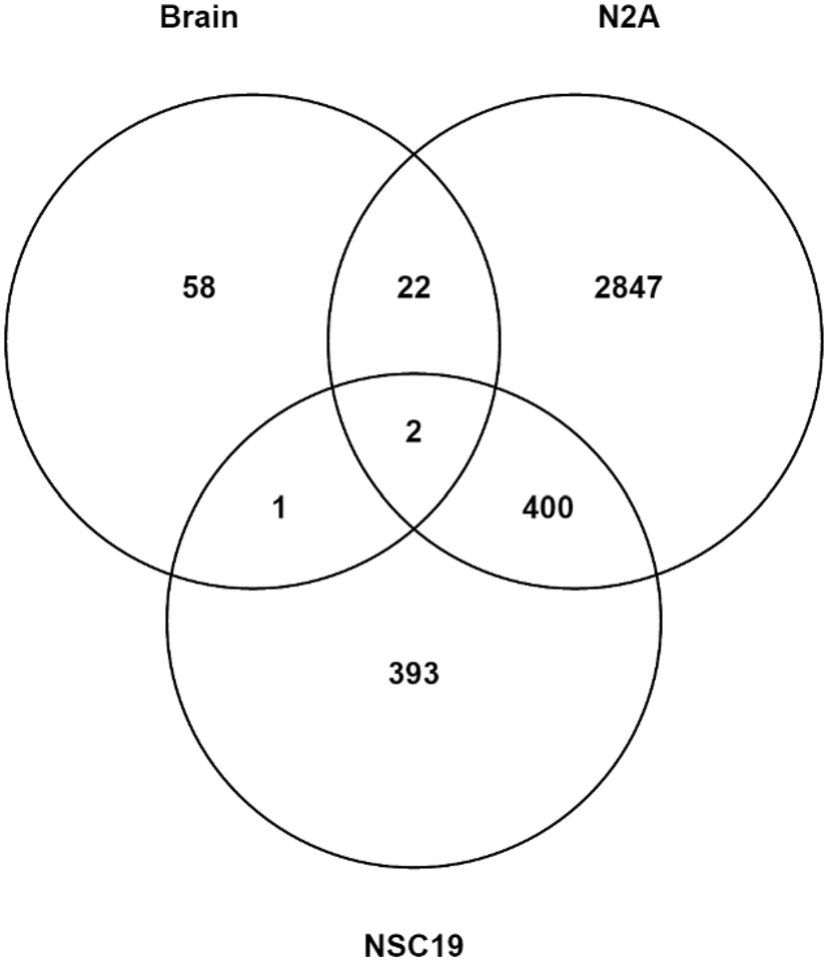
Venn diagram of genes called significantly regulated (*q* < 0.2) in microarray analysis of mouse brain, N2A cells and NSC19 cells exposed to HBCD. Mice were exposed to HBCD via the diet for 28 days at a dose of 199 mg/kg body weight per day, resulting in a HBCD concentration in the brain of 40 µmol/kg (wet weight). N2A and NSC19 cells were exposed to either 1 or 2 µM of HBCD for 24 h and the numerals shown are the combined numbers of unique genes called significant in the two conditions. The full lists of differentially regulated genes, fold-change data and associated p- and FDR can be found in Supplementary Table 2. The Venn diagram was constructed using Venny (http://bioinfogp.cnb.csic.es/tools/venny/index.html)

Fold-change data, associated *p*- and FDR-values for all statistically analysed and mapped genes are presented in the supplementary material (Supplementary Table 2). In all conditions, except one (N2A, 1 µM), there were a greater number of genes that were downregulated than upregulated (Table 1). In mice exposed to HBCD through the diet (199 mg/kg bw/day) a total of 83 genes were differentially regulated, of which 10 were upregulated and 73 downregulated. The greatest number of regulated genes were observed in N2A cells treated with 2 µM HBCD. HBCD elicited distinct and overlapping responses in gene expression in vivo and in vitro (Fig. 2, Table 2). In total, 25 (30%) of the genes that were regulated in mouse brain were also regulated in either or both of the cell lines (Fig. 2). The identity and expression pattern of these are shown in Table 2. The 30% overlap between genes regulated in mouse with those regulated in the cell lines gives confidence in the analysis. The microarray results were further verified by qPCR analysis (Supplementary Table 3). Out of 15 genes found significantly regulated on microarrays, 14 were also significantly regulated when analysed by qPCR although directionality of the response was not always the same in all conditions.

**Table 1:** Number of differentially expressed genes (FDR 20%) in brains of mice, N2A cell line, and NS19 cell line, following exposure to HBCD

Analysis	Concentration (µM)	Numbers of regulated genes	Up regulation	Down regulation
Brain	7.9^a^	83	10	73
N2A	1	27	22	5
N2A	2	3265	1599	1666
NSC19	1	796	342	454
NSC19	2	356	157	199

Mice were exposed to a daily oral HBCD dose of 199 mg/kg for 28 days and cell lines were exposed for 24 h. The exposure concentration indicated for brain is that measured in brain tissue

^a^Concentration in brain tissue (µmol/kg wet weight). Mice were exposed to HBCD in the diet for 28 days at a daily dose of 199 mg/kg body weight

**Table 2:** Genes differentially expressed in mouse brain and cell lines

Gene symbol	Entrez gene name	Log_2_ ratio (brain)	FDR (brain)
*Brain+N2A+NSC19*			
Etv5	ets variant 5	− 0.340	0.122
Yars2	Tyrosyl-tRNA synthetase 2, mitochondrial	− 0.328	0.048
*Brain+NSC19*			
Cmc2	C-x(9)-C motif containing 2	− 0.280	0.050
*Brain+N2A*			
Cdkn1a	Cyclin-dependent kinase inhibitor 1A (p21, Cip1)	− 0.570	0.050
Hsp5a	Heat shock 70 kDa protein 5 (glucose-regulated protein, 78 kDa)	− 0.434	0.066
Fosb	FBJ murine osteosarcoma viral oncogene homolog B	− 0.406	0.187
Vgf	VGF nerve growth factor inducible	− 0.344	0.050
Mrpl13	Mitochondrial ribosomal protein L13	− 0.318	0.050
Syt12	Synaptotagmin XII	− 0.288	0.122
Snx16	Sorting nexin 16	− 0.272	0.048
Txnip	Thioredoxin interacting protein	− 0.264	0.156
St8sia5	ST8 alpha-N-acetyl-neuraminide alpha-2,8-sialyltransferase 5	− 0.258	0.010
Xbp1	X-box binding protein 1	− 0.248	0.135
Ppp4c	Protein phosphatase 4, catalytic subunit	− 0.244	0.120
Pdia6	Protein disulfide isomerase family A, member 6	− 0.242	0.172
Fuca1	Fucosidase, alpha-l-1, tissue	− 0.238	0.037
Ostc	Oligosaccharyltransferase complex subunit (non-catalytic)	− 0.238	0.135
Pdp1	Pyruvate dehyrogenase phosphatase catalytic subunit 1	− 0.222	0.174
Fscn1	Fascin actin-bundling protein 1	− 0.220	0.048
Otub2	OTU deubiquitinase, ubiquitin aldehyde binding 2	− 0.218	0.105
Abhd6	Abhydrolase domain containing 6	− 0.204	0.073
Ddx52	DEAD (Asp-Glu-Ala-Asp) box polypeptide 52	− 0.202	0.081
Ndufb2	NADH dehydrogenase (ubiquinone) 1 beta subcomplex, 2, 8 kDa	− 0.200	0.084
Coq4	Coenzyme Q4	0.204	0.119
Cacfd1	Calcium channel flower domain containing 1	0.232	0.130

### Pathway analysis of transcriptomics data

**Fig. 3:**
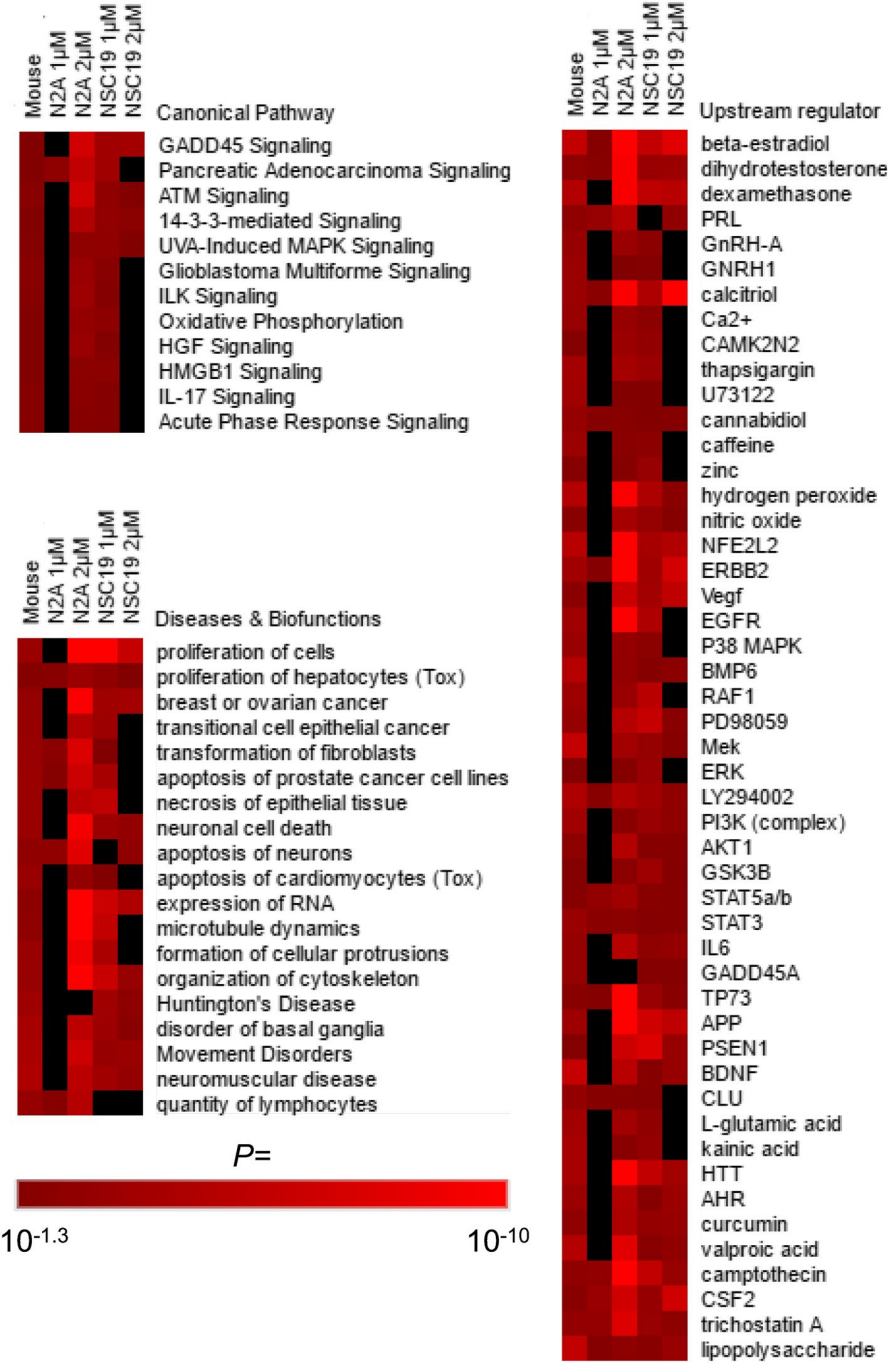
Pathway analysis of genes called significantly regulated (*q* < 0.2) in microarray analysis of mouse brain, N2A cells and NSC19 cells exposed to HBCD. Mice were exposed to HBCD via the diet for 28 days at a dose of 199 mg/kg body weight per day, resulting in a HBCD concentration in the brain of 40 µmol/kg (wet weight). N2A and NSC19 cells were exposed to either 1 or 2 µM of HBCD for 24 h. Lists of significantly regulated genes from each condition were subjected to pathway analysis, using the IPA software, and statistical significance of overrepresentation of genes in different “Canonical Pathways” and “Diseases & Biofunctions” are shown as a heat-map along with predicted “Upstream regulators”. Only selected pathways that had significant enrichment in mouse brain plus two cell culture conditions are shown. The full data-set including genes in each pathway is presented in Supplementary Tables 4A–D

Significantly regulated genes (FDR < 20%) were subjected to targeted biological network analysis on the Ingenuity^®^ Pathway Analysis (IPA^®^) platform (Supplementary Table 4A-D). Categories with < 3 genes were removed and non-redundant categories that were significantly enriched (*p* < 0.05) in brain and at least two cell culture conditions are shown in Fig. 3.

*Canonical pathways* for ‘GADD45 Signaling’, ‘ATM Signaling’, ‘Pancreatic Adenocarcinoma Signaling’, ‘ATM Signaling’, ‘14-3-3-mediated Signaling’ and UVA-Induced MAPK Signaling’ were significantly affected in four out of the five conditions investigated (Fig. 3). Enrichment testing of the gene-lists against ‘*Diseases & Functions*’ annotations revealed that HBCD caused differential expression of genes preferentially related to (1) cell proliferation, (2) cancer, (3) apoptosis, (5) cytoskeleton, and (4) neuromuscular diseases (Fig. 3).

*Upstream regulator* analysis in IPA^®^ is a prediction of the transcriptional cascade based on the number of targets of “transcriptional regulators” in the dataset compared with those in the IPA^®^ database. Broadly, the implicated upstream regulators may be divided into (1) steroid and sex hormones, (2) calcium and zinc regulation-related, (3) kinase cascades, (4) cytokine and growth-factor response, (5) neurodegenerative disease, (6) and xenobiotic response. Genes in transcriptional networks known to be regulated by ‘beta-estradiol’, ‘dihydrotestosterone’, ‘PRL’ (prolactin), ‘calcitrol’ (1,25-dihydroxycholecalciferol), ‘ERBB2’ (Erb-B2 Receptor Tyrosine Kinase 2; aka HER2), ‘camptothecin’, ‘CSF2’ (Colony Stimulating Factor 2), ‘trichostatin A’, ‘lipopolysaccharide’, ‘STAT3’ (Signal transducer and activator of transcription 3), ‘STAT5a/b’, ‘TP73’ (Tumor Protein P73) were enriched in all five conditions, implicating these as key networks explaining the responses to HBCD (Fig. 3). The prediction of ‘l-glutamic acid’ and ‘kainic acid’ as upstream regulators is also interesting as it implicates specific effects on glutamatergic neurons.

Upon examining the prediction of upstream regulators by IPA^®^ the large numbers of networks known to be influenced by and/or interfering with intracellular Zn^2+^ and Ca^2+^ signals was striking (Fig. 3). These also included ‘zinc’ and ‘Ca^2+^’ themselves. Furthermore, glutamatergic receptor activation, as implicated through ‘l-glutamic acid’ and ‘kainic acid’ as upstream regulators, results in a post-synaptic rise in intracellular [Ca^2+^], an effect that in many neurons is modulated my Zn^2+^ (Marger et al. 2014; Sensi et al. 2011; Sunuwar et al. 2017). The predominance of Zn^2+^ and Ca^2+^ regulated networks prompted us to further investigate the effects of HBCD on neuronal Zn^2+^ and Ca^2+^ signalling, focussing on glutamatergic neurons.

### HBCD-evoked Zn^2+^ signals in N2A cells

**Fig. 4:**
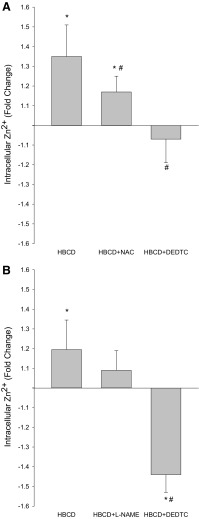
Intracellular [Zn^2+^] in N2A cells exposed to HBCD for 24 h. N2A cells were exposed to HBCD with or without other treatments for 24 h and intracellular Zn^2+^ release was detected using the Zn^2+^-specific fluorescent probe, Zinpyr-1. **a** N2A cells were incubated for 24 h with 1 µM HBCD with or without 2 h pre-incubation with the antioxidant, NAC, or the zinc chelator, DEDTC. **b** The N2A cell line was incubated for 24 h with 1 µM HBCD with or without 2 h pre-incubation with the NO synthase blocker, L-NAME or with DEDTC. The experiment was carried out three times with 16 technical replicates in each experiment (*n* = 3). Data are expressed as fold-change of fluorescence measured in the control. The bars represent the averages of values from the three experiments and error bars show the standard deviation. An asterisk and a hash sign indicate a statistical differences from untreated control cells and cells treated with 1 µM HBCD, respectively (ANOVA, *p* < 0.05)

The potential of HBCD to trigger Zn^2+^ signals was investigated in N2A cells using the Zn^2+^-specific probe, Zinpyr-1. For this experiment whole cell population fluorescence was recorded as a relative cumulative measure of intracellular [Zn^2+^]_i_ and we found that exposure of cells to 1 µM HBCD caused on average a 1.2–1.4-fold increase in [Zn^2+^]_i_ (*p* < 0.05; Fig. 4a, b). Thus, HBCD does evoke Zn^2+^ signals in N2A cells.

Application of 1 µM of the membrane permeable zinc chelator, diethyldithiocarbamate (DEDTC) completely abolished the Zn^2+^ signal, confirming that fluorescence was caused by intracellular Zn^2+^ release (Fig. 4a, b). Zinc is released from metallothioneins and other proteins in cells by an increase in the redox potential (Berendji et al. 1997; Chung et al. 2006). Therefore, 1 µM *N*-acetyl cysteine (NAC) was added to the culture medium to test if the Zn^2+^ release was caused by HBCD-induced oxidative stress. NAC is a substrate for biosynthesis of reduced glutathione, which is the most abundant antioxidant in cells (Kerksick and Willoughby 2005). Addition of NAC partially alleviated HBCD-induced Zn^2+^ release compared with 1 µM HBCD alone, suggesting that oxidative stress was involved (Fig. 4a). Since nitric oxide (NO) pathway activation is a main cause of oxidative stress damage in cells, proteins and lipids (Chung 2010), we hypothesised that HBCD might activate nitric oxide synthase, leading to NO production and nitrosylation of zinc thiolate bonds in metallothioneins, leading to Zn^2+^ release. We, therefore, investigated the Zn^2+^ concentration in cells exposed to HBCD following treatment with the NO pathway blocker L-NAME. Again, HBCD triggered intracellular release of Zn^2+^, but contrary to our hypothesis treatment of cells with L-NAME did not reduce the HBCD evoked Zn^2+^ signal, indicating that the effect is not related to NOS activation (Fig. 4b). In this experiment, the release of Zn^2+^ following HBCD treatment alone was slightly but not significantly lower than that shown in Fig. 4a, however, the reversal of this signal by DEDTC was more substantial than in the experiment described in Fig. 4a and 1.4-fold below the Zinpyr-1 fluorescence in control cells. Such quantitative differences between experiments can be caused by variability in Zinpyr-1 and/or DEDTC loading. Importantly, the significant increase of intracellular Zn^2+^ by HBCD and the reversal of this signal by DEDTC were maintained in both set of experiments. Thus, it can be concluded that HBCD causes intracellular Zn^2+^ release through oxidative stress, but this is not a result of nitric oxide synthase activation.

### HBCD inhibition of metabotropic Ca^2+^ signals in primary neuronal cultures

**Fig. 5:**
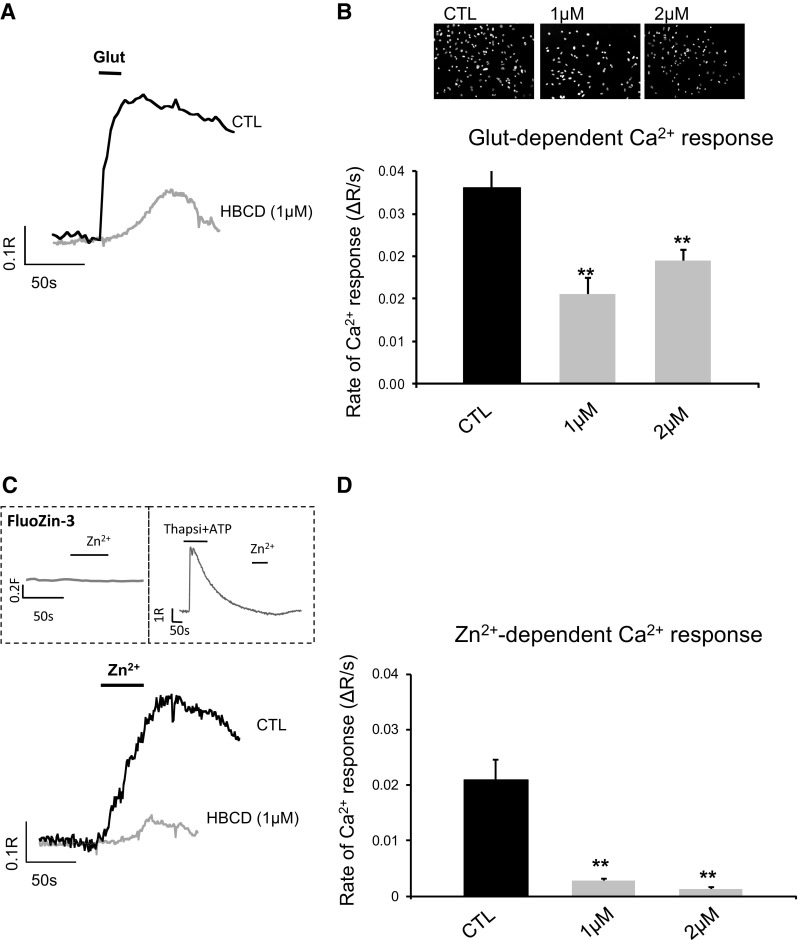
HBCD effects on glutamate- and zinc-dependent Ca^2+^ transients. Primary hippocampal mixed neuronal cultures were exposed to 1 or 2 µM of HBCD for 24 h. Cells were loaded with Fura-2 AM and intracellular Ca^2+^ responses to (**a**, **b**) glutamate (200 µM, 30 s) or (**c**, **d**) Zn^2+^ (200 µM, 30 s) were monitored. A representative Ca^2+^ response (**a**, **c**) and the averaged initial rates of fluorescence change (**b**, **d**) are shown. Note that the Ca^2+^ response is monitored after a short lag time following the application of the ligand, likely due to the timing of the perfusion system. The initial rate of the response is dependent on the net influx of Ca^2+^ into the cytoplasm and represents the activity of GPR39. Inserts to **b** show representative DAPI staining of neuronal cultures, seeded with the same number of cells, following control (DMSO alone) or HBCD treatment at the indicated concentration. Inserts to **c** show: left panel: neurons loaded with the Zn^2+^ sensitive dye, Fluozin-3 AM, treated with Zn^2+^ (200 µM, 30 s) as marked by the bar; right panel: Neurons loaded with Fura-2 AM, treated with Thapsigargin (200 nM) and ATP (5 µM) as marked, and subsequent to the depletion of Ca^2+^ stores and recovery of the fluorescent signal to baseline, Zn^2+^ (200 µM, 30 s) was applied as marked. The bars represent the arithmetic mean of three independent experiments, each consisting of at least 7 averaged replicates for each condition (*n* = 3), **p < 0.01

Many glutamatergic neurons co-release glutamate and Zn^2+^ (Sensi et al. 2011; Sunuwar et al. 2017; Marger et al. 2014). To determine the effect of HBCD treatment on signalling capacity of cells in primary neuronal cultures, we wanted to determine the glutamate and extracellular Zn^2+^ dependent Ca^2+^ responses triggered via metabotropic receptors (Besser et al. 2009; Ganay et al. 2015; Kato et al. 2012). We initially asked what is the concentration of HBCD that is toxic to primary neurons. Hippocampal neuronal cultures were treated with HBCD at 1, 2, or 4 µM of HBCD for 24 h and control neurons were treated with the solvent (DMSO). We then fluorescently monitored the Ca^2+^ responses in Fura-2 loaded cells by single cell recording. In control cells, application glutamate (200 µM) triggered a rapid rise of Fura-2 fluorescence (about 60–70% increase above baseline fluorescence; Fig. 5a, b), as previously described for these metabotropic intracellular Ca^2+^ responses (Besser et al. 2009; Ganay et al. 2015). The perfusing extracellular solution was Ca^2+^-free, thus allowing measurements of the initial metabotropic response triggered to release Ca^2+^ from endoplasmic reticulum stores. Following HBCD treatment, cultures were stained with DAPI and as shown in the inserts Fig. 5b, cell survival was slightly reduced, however, Fura-2 analysis is performed only on the surviving neurons, which are able to induce esterase-dependent cleavage of the AM group. Hence although some of the cells did not survive, we were able to perform the analysis even on cultures treated with 2 µM of HBCD. Interestingly, treatment of the cultures with 1 µM of HBCD caused an approximately 50% decrease in the glutamate-dependent Ca^2+^ signalling in viable neurons, but no further reduction was observed at higher concentrations (Fig. 5a, b). We then asked if the role of Zn^2+^ and Ca^2+^ highlighted by the network analysis may represent a change in the response of the ZnR/GPR39, a metabotropic pathway that senses changes in extracellular Zn^2+^ to trigger Ca^2+^ signalling (Besser et al. 2009; Ganay et al. 2015). We, therefore, applied Zn^2+^ as was previously shown to activate the ZnR/GPR39 (Chorin et al. 2011; Saadi et al. 2012). Indeed, as shown in the inserts of Fig. 5c, an intracellular Zn^2+^ sensitive dye, Fluozin-3, did not exhibit a change of signal in hippocampal neurons following administration of Zn^2+^ (200 µM) under the same conditions used to trigger ZnR/GPR39 signalling (dashed top-left panel). In the control hippocampal cells (Fig. 5c) Zn^2+^ (200 µM) triggered a rapid rise of Fura-2 fluorescence (about 60–70% increase above baseline fluorescence), similar to the response to glutamate (Fig. 5a). In contrast, following emptying of intracellular Ca^2+^ stores, using Thapsigargin (200 nM) and ATP (5 µM), we did not detect an increase in the signal of the Ca^2+^ sensitive dye Fura-2 (Fig. 5c, top-right insert). Thus, we conclude that the application of Zn^2+^, at concentrations and times used here, induces metabotropic signalling in hippocampal neurons as we have shown previously (Ganay et al. 2015; Saadi et al. 2012). We then studied the effect of HBCD on the extracellular Zn^2+^ dependent Ca^2+^ signalling. The Zn^2+^-dependent Ca^2+^ response in cells treated with HBCD was abolished resulting in about 90% inhibition of the signal by all concentrations of HBCD tested (Fig. 5c, d). The lower initial rates of the response to both glutamate and Zn^2+^ suggest that metabotropic signalling is impaired by the HBCD treatment. This may result from partial depletion of the intracellular Ca^2+^ stores as well as changes in the functional properties or expression of the metabotropic receptors. Hence, while HBCD treatment yielded a small, albeit clear, metabotropic response to glutamate, the response to Zn^2+^ was largely absent in the treated cells.

## Discussion

Omics technologies enable experiments that can implicate molecular networks affected by toxicants and shed light on key events in adverse outcome pathways (AOPs) (EFSA 2014). Because omics profiling can be carried out without prior hypothesis, they can be considered unbiased and discovery-driven. In the present study, we sought to characterise robust transcriptomic responses to HBCD by combining gene expression profiles resulting from in vivo and in vitro exposures. We compared gene expression profiles in brain of juvenile mouse orally exposed for 28 days to HBCD to those in two neuronally derived cell lines exposed to HBCD for 1 day, and were able to implicate a number of gene networks that were affected both in vivo and in vitro. These gene sets included networks previously identified as being targeted by HBCD (hypothalamus-pituitary–gonadal axis; Ca^2+^ signalling), but interestingly also shed light on several new pathways that were affected. Some of the more notable of these were pathways involved in DNA repair and cell proliferation, and transcriptional networks activated by zinc signals, tyrosine kinase receptors, inflammation, and reactive oxygen species.

A few previous studies have focussed on transcript and protein profiling in the liver of rodents and zebrafish treated with HBCD, and these collectively provide very strong evidence for interference with basic metabolic pathways (gluconeogenesis/glycolysis, amino acid/protein metabolism, lipid metabolism) and stress responses (Canton et al. 2008; Kling and Forlin 2009; Miller et al. 2016). However, cultured human cell lines from lung (A549) and liver (HepG2/C3A) showed few changes in global transcript and metabolite profiles in response to HBCD concentrations up to 2 µM (Zhang et al. 2015). Oral exposure of female Balb/c mice to HBCD with the same exposure regime and conditions as in the present study was previously found by our team to result in differential expression of 90 genes and 10 proteins (Rasinger et al. 2014). The list of differentially expressed genes in our previous study was particularly enriched for olfactory receptors, G-coupled proteins, and protein tyrosine phosphatases (Rasinger et al. 2014). In the present study, the number of differentially regulated genes in mouse brain following HBCD exposure (83 genes) was very similar to that in our earlier experiment (90 genes). Many of the genes found to be regulated in our previous study were also differentially expressed in mouse brain and neuronal cell lines in the present study.

IPA analysis revealed a strong inference of neuronal dyshomeostasis of Ca^2+^ and Zn^2+^ and implication of glutamatergic neurons being affected. We carried on with mechanistic studies in cultured cells to show that HBCD does indeed disrupt both Ca^2+^ and Zn^2+^ signalling in neurons. Effects of HBCD on calcium homeostasis have been described before in cell lines of neuronal and cardiac origin (Al-Mousa and Michelangeli 2014; Dingemans et al. 2009; Wu et al. 2016b) and are now corroborated by in vivo evidence, based on transcriptional networks in mouse brain, and targeted experimental information on reduced Ca^2+^ transients in response to glutamate in isolated hippocampal neurons. The demonstration of HBCD disruption of neuronal Zn^2+^ homeostasis is completely novel, although this mechanism of action has been proposed for triclosan effects on rat thymocytes (Tamura et al. 2012) and 2,3,7,8-tetrachlorodibenzodioxin effects on brain (Rasinger et al. 2014). Disruption of cellular Zn^2+^ homeostasis is also recognised as a mechanism of action for soft Lewis acid transition element, such as cadmium and mercury (Kawanai et al. 2009; Predki and Sarkar 1994).

Our understanding of and appreciation for the roles of zinc in biology and disease of the brain has been hugely increased in recent years (Marger et al. 2014; McCord and Aizenman 2014; Sensi et al. 2011; Tamano et al. 2016). Deficiency or excess of Zn^2+^ can cause alteration in behaviour, abnormal central nervous system development, and neurological disease (Bitanihirwe and Cunningham 2009). In the brain, Zn^2+^ modulates neuronal post-synaptic potentials and synaptic plasticity, and is implicated in the processes of learning and memory (Marger et al. 2014; Tamano et al. 2016). The vast majority of zinc in cells including neurons is bound to metalloproteins, but ionic Zn^2+^ is concentrated in synaptic vesicles of a sub-set of glutamatergic neurons (also known as zincergic neurons) (Sensi et al. 2011). Synaptic Zn^2+^ is co-released with glutamate and acts as neuromodulator of *N*-methyl-d-aspartate (NMDA) receptors (Anderson et al. 2015; Pan et al. 2011; Qian and Noebels 2005; Sensi et al. 2011; Takeda 2000) or AMPA receptors (Kalappa et al. 2015). The GluN2A subunit of the NMDA receptor has a high affinity Zn^2+^-binding site that mediates allosteric inhibition of post-synaptic currents (Sensi et al. 2011). There is also evidence for a physiological role of Zn^2+^ inhibition of γ-amino butyric acid-ergic (GABAergic) transmission via blockage of T-type Ca^2+^ channels (Grauert et al. 2014). Finally, Zn^2+^ can evoke post-synaptic currents via activation of GPR39, which is a metabotropic zinc receptor that stimulates Ca^2+^ release in cells (Besser et al. 2009) and regulates neuronal excitability (Chorin et al. 2011; Gilad et al. 2015; Saadi et al. 2012). In the present study, we show that HBCD depresses glutamate and zinc evoked Ca^2+^ transients in hippocampal neurons that survived the HBCD treatment and were able to hydrolyse the Fura-2 AM to allow the measurement. Inactivation of SERCA by HBCD has been demonstrated, and this may lead to some depletion of the cytosolic Ca^2+^ stores. However, we show that the inhibitory effect is stronger in Zn^2+^ treated cells compared to the glutamate treatment, suggesting that there is also a direct effect on the metabotropic signalling pathway and not only on the Ca^2+^ stores.

HBCD also stimulates intracellular Zn^2+^ release in N2A cells. The ability of NAC to partially alleviate the HBCD-evoked Zn^2+^ signal confirms that low levels of HBCD can generate oxidative stress (Deng et al. 2009). However, this effect was not caused by NOS activation as L-NAME did not influence Zn^2+^ release. In light of the physiological roles of synaptic Zn^2+^ in neural transmission and specific functions in mediating synaptic plasticity for learning and memory or cell death, disruption in neuronal zinc homeostasis could be causing adverse apical effects. For example, exposure to 1 µM HBCD activated caspase-3 activity in N2A and NSC19 cells (Fig. 1c, d) and microarray analysis revealed that apoptotic gene networks were affected in brain of HBCD exposed mice as well as in both neuronal cell lines (Fig. 3). Moreover, Zn^2+^ and Ca^2+^ cooperate in mediating neuronal apoptosis in response to ischemia, oxidative stress, and other forms of injury (McCord and Aizenman 2013, 2014). This interaction converges on the voltage-gated, delayed rectifier K^+^ channel Kv2.1, which when activated and residing in the plasma membrane allows the efflux of K^+^ required for apoptosis (McCord and Aizenman 2013, 2014). Kv2.1 is activated through phosphorylation by p38 MAPK and tyrosine kinase Src. Both these kinases are activated by intracellular Zn^2+^ signals (Hogstrand et al. 2009; McCord and Aizenman 2013), and p38 MAPK was a predicted upstream regulator in HBCD exposed mouse brain, N2A cells and NSC19 cells in the present study. Translocation of Kv2.1 from endosomes to the plasma membrane where it is active is mediated by syntaxin SNARE proteins downstream CaMK2 activation (McCord and Aizenman 2013). Interestingly, we found in our previous study that CaMK2 expression is regulated in mouse brain during HBCD exposure (Rasinger et al. 2014) and in the present study the CaMK2 inhibitory protein, CaMKN2, was a predicted upstream regulator of gene expression. Furthermore, the transcript for its paralogue, CaMKN1, was significantly regulated in both cell lines following exposure to 2 µM HBCD (Supplementary Table 2). We, therefore, hypothesise that apoptosis in brain of HBCD exposed mice was triggered by the disruption of Ca^2+^ and Zn^2+^ homeostasis.

Prediction of upstream regulators following HBCD exposure implicated gene networks in the hypothalamus-hypophysis-gonadal axis (GnRH1, 17ß-oestradiol, dihydrotestosterone) as well as prolactin (PRL) with regulation by 17ß-oestradiol and dihydrotestosterone being statistically significant in mouse brain, both cell lines, and both doses (Fig. 3; Supplementary Table 4B). This finding is in line with earlier findings that HBCD has endocrine disruption potential (Hamers et al. 2006; Krivoshiev et al. 2016; Maranghi et al. 2013; van der Ven et al. 2009). Developmental exposure of Wistar rats to HBCD resulted in reduced testicular size with a lower confidence limit for a 5% effect of as little as 11.5 mg/kg body weight per day (van der Ven et al. 2009). Our team has previously observed an increased serum testosterone concentration and testosterone/17ß-oestradiol ratio in female Balb/c mice undergoing the same HBCD exposure regime as that used in the present study [199 mg/kg body weight per day for 28 days; (Maranghi et al. 2013)]. In the present study, HBCD effects on oestrogen and androgen gene networks were observed not only in vivo but also on two different neuronal cell lines, strongly suggesting direct effects on sex steroid target tissue. HBCD showed both anti-androgenic and anti-oestrogenic activity in CALUX^®^ sex-steroid receptor reporter gene assays and anti-oestrogenic activity in a MCF-7 cell proliferation assay (Hamers et al. 2006; Krivoshiev et al. 2016). This suggests that HBCD may bind to and disable sex-steroid nuclear receptors and would be consistent with animal data from the present and previous studies (Maranghi et al. 2013; van der Ven et al. 2009).

## Electronic supplementary material

Below is the link to the electronic supplementary material.

Supplementary material 1 (XLSX 1433 kb)
